# The portable wearBands resistance system increases exercise intensity during mixed-reality boxing without reducing enjoyment or flow

**DOI:** 10.3389/fspor.2026.1938500

**Published:** 2026-09-02

**Authors:** Jacek Polechoński, Aleksandra Daniel, Małgorzata Dębska-Janus, Anna Witkowska, Jarosław Cholewa

**Affiliations:** 1 Institute of Sport Sciences, Academy of Physical Education in Katowice, Katowice, Poland; 2 Student Research Society for Physical Activity and Tourism in Virtual Reality “ACTIVE VR”, Academy of Physical Education in Katowice, Katowice, Poland

**Keywords:** boxing, elastic resistance, enjoyment, exercise intensity, flow, mixed reality, physical activity, WearBands

## Abstract

**Introduction:**

Physical activity in a mixed-reality (MR) environment may be an attractive exercise; however, its intensity depends on application characteristics and the extent of whole-body involvement. One method of increasing exercise load during activities based primarily on upper-limb movements may be additional elastic resistance. The aim of this study was to assess the effects of elastic resistance applied to the upper limbs on exercise intensity, perceived exertion, enjoyment, and flow state during boxing in MR.

**Method:**

The study included 36 university students, comprising 20 women (age: 22.25 ± 1.52 years) and 16 men (age: 22.00 ± 1.46 years). Each participant completed three 10-minute simulated boxing bouts against an artificial intelligence-controlled opponent in the Golden Gloves application using a Meta Quest 3 headset in mixed-reality mode. Trials were performed without resistance and with lower or higher elastic resistance generated on the participant's body using the WearBands system. The order of conditions was counterbalanced. Exercise intensity was assessed based on the percentage of maximum heart rate (%HR_max_) and the Borg 6–20 scale. Enjoyment was assessed using the short Physical Activity Enjoyment Scale, whereas flow state was evaluated using the short Flow State Scale.

**Results:**

Increasing elastic resistance resulted in a significant increase in mean heart rate, from 156.61 ± 15.26 beats/min without resistance to 164.97 ± 13.99 beats/min with lower resistance and 169.75 ± 13.42 beats/min with higher resistance (*p* < 0.001; η^2^p = 0.63). A similar relationship was found for %HR_max_, which increased from 81.34 ± 7.83% to 85.68 ± 7.21% and 88.17 ± 6.96%, respectively (*p* < 0.001; η^2^p = 0.63). Perceived exertion also increased with resistance, reaching 10.86 ± 2.32, 13.50 ± 2.13, and 15.83 ± 1.84 points, respectively (*p* < 0.001; W = 0.84). No significant differences were found in enjoyment or flow state.

**Discussion:**

The portable elastic resistance system for upper limbs progressively increased heart rate-based and perceived intensity during a 10-minute MR boxing bout without reducing enjoyment or flow in active university students. These findings suggest WearBands may practically manipulate MR exercise load; longitudinal studies are needed to assess safety, adherence, metabolic demand, and training effects.

## 1 Introduction

The dynamic development of immersive technologies has moved digital environments beyond passive entertainment and into physical activity (PA), sports training, motor education, and rehabilitation. Immersive virtual reality (VR) uses a head-mounted display to present a computer-generated environment, that users interact with through head, trunk, and limb movements. Compared with screen-based games, VR permits spatial motion tracking, real-time feedback, and a stronger sense of presence. Consequently, active video games (AVGs) and VR applications may support PA promotion, health-oriented and sports training, motor education, and therapy (1–3).

The range of movement-related VR applications is broad. This technology is used to reproduce and teach complex sports activities, assess reaction time, analyze movement, develop balance and functional fitness, and conduct both aerobic and resistance exercise. Research has indicated, among other things, the potential to improve reactive behaviors in combat sports, transfer some of the skills acquired in virtual environments to real-world conditions, and effectively conduct training using ergometers, omnidirectional treadmills, and AVGs (4–7). The literature has evaluated not only the effectiveness of such interventions, but also their physiological intensity, perceived exertion, exercise enjoyment, motivation, engagement, and flow state (1, 2, 8). This indicates that the evidence base concerning PA in VR is now relatively extensive, although it remains heterogeneous in terms of the equipment used, application type, population, and measurement methods.

VR can support engaging tasks within limited space, graded difficulty, immediate feedback, repeatable conditions, and training situations that costly, difficult, or dangerous to organize in the real word. It may also divert attention from unpleasant somatic sensations, but effects depend on the application, immersion, task, and user characteristics (2, 9, 10). A key limitation of fully immersive VR is loss of visual access to the real environment, which may increase collision risk during dynamic exercise and requires a suitable safety boundary. Additional limitations include cybersickness, headset-related burden, and incomplete correspondence between real and digitally represented movements (11, 12).

Some limitations of fully immersive VR may be reduced through mixed reality (MR), which combines real and digital elements along the reality–virtuality continuum (13). Contemporary consumer headsets can provide a full-color, low-latency view of the surrounding environment while spatially registering virtual objects within it (14).

A significant milestone in the popularization of this form of interaction was the introduction of wireless consumer headsets equipped with full-color passthrough, automatic room mapping, and depth sensors. Meta Quest 3, launched in 2023, was not the first MR device, but it substantially lowered the barrier to accessing this type of experience and improved its usability in home settings. Through outward-facing cameras, users can see their own body, the floor, walls, furniture, and nearby people, while an opponent, targets, or other training objects are superimposed onto the real-world space. Research on the use of passthrough mode indicates that maintaining visibility of the surrounding environment may improve spatial orientation and task performance without requiring a complete loss of the sense of presence within the digital environment (15).

From the perspective of physical activity, MR allows users to monitor their position relative to obstacles, use real-world accessories, and combine digital cues with a partner or instructor. It may therefore support training that is more closely integrated with real-world movement and equipment. Nevertheless, the passthrough view remains camera-mediated, may involve latency and distortion, and does not eliminate collision risk. MR has also been studied far less extensively than VR in the context of physical exercise, partly because appropriate consumer hardware and dedicated applications have become available only relatively recently (12, 14).

Most popular VR and MR headsets primarily track the position of the head and handheld controllers, favoring applications based on upper-limb movements. Active games therefore include racket-sport simulations and boxing applications, in which users may strike targets, work with virtual pads or bags, perform prescribed combinations, or fight an artificial intelligence-controlled opponent. Some applications originally designed for fully immersive VR now also offer an MR mode, in which the opponent and interface elements appear within the real room.

Boxing is a particularly interesting form of digital physical activity because it requires rapid, multidirectional arm movements, trunk rotations, evasive maneuvers, stance changes, and responses to an opponent. The extent of lower-limb involvement, however, depends on application mechanics, available space, and user behavior. When real whole-body movement is required, such as walking on an omnidirectional treadmill, heart rate, metabolic cost, and perceived exertion are greater than with movement controlled by handheld controllers (7); arm-dominant games may otherwise reach only low or moderate intensity (16, 17).

For this reason, attempts have been made to increase the physiological load during immersive active games without the need to use expensive and space-consuming exercise devices. One simple solution is to apply additional loading to the limbs. In a study using Beat Saber, the use of wrist weights increased exercise intensity from a low to a moderate level without causing a significant deterioration in user comfort (16). In a study involving locomotion on an omnidirectional treadmill, weights attached at the ankles further increased the already high intensity of the activity and did not reduce game enjoyment (18). These findings confirm that appropriately selected external resistance may increase the physiological cost of exercise in a virtual environment; however, the type of resistance should be matched to the dominant movement patterns and must not interfere with control or safety.

An alternative to fixed weights is elastic resistance, the magnitude of which increases as the elastic band is stretched. This solution may be better suited to dynamic boxing movements because resistance increases during punch execution and decreases once the punch has been completed. In a study using the BoxVR application, elastic bands attached to the distal parts of the upper limbs and anchored externally to the body increased exercise intensity; low resistance did not reduce enjoyment, whereas higher resistance led to a decrease in enjoyment (19). However, such a configuration has a functional limitation: a fixed external anchoring point restricts freedom of movement and may alter the direction of force application during rotational movements. For this reason, it is primarily suitable for tasks performed in a fixed position, whereas it is less useful in a realistic bout in which the participant should be able to freely change position, perform evasive movements, and move relative to the opponent.

A solution that preserves mobility is provided by wearable resistance band systems, in which elastic elements are anchored directly to the exerciser's body. One example is WearBands, which consists of a hip belt, elastic resistance tubes, and attachments placed on the limbs. By connecting anchoring points on the belt and the limbs, resistance is generated during movement without the need to attach the bands to a wall, floor, or device. The user can therefore move, rotate, and change direction while maintaining resistance on the limbs. The system has been used in plyometric training in young tennis players, in which additional resistance was incorporated into jumping and multidirectional exercises (20). Although this does not allow direct conclusions to be drawn regarding its effectiveness during MR boxing, it confirms that WearBands can be used in dynamic sports tasks requiring freedom of movement.

The assessment of the usefulness of additional resistance during immersive activity should not be limited to physiological indicators. Exercise may be objectively intense while simultaneously being perceived as excessively strenuous, unpleasant, or disruptive to task performance. Conversely, the digital environment, narrative, and focus on the opponent may partly distract the participant from fatigue-related sensations. Studies have demonstrated discrepancies between objective and subjective assessments of exertion during active VR games, which supports the simultaneous use of heart rate monitoring and a perceived exertion scale (17). Rating of perceived exertion (RPE) reflects the participant's overall interpretation of the exercise load and may respond not only to muscular and cardiorespiratory demands, but also to equipment-related discomfort, task complexity, and competition-related stress.

Enjoyment of the activity is equally important because positive exercise experiences are associated with the intention to exercise again and may promote long-term adherence. Reviews indicate that immersive VR often increases enjoyment, intrinsic motivation, and positive affective responses, although the magnitude of these effects depends on the application and the comparison condition (1, 2). Experimental studies have shown, among other findings, greater enjoyment and more favorable psychological responses during interactive VR exercise than under less engaging screen-based conditions (8). At the same time, excessive resistance may impair the experience even when it effectively increases heart rate; therefore, determining the relationship between load magnitude and the perceived quality of training is of practical importance (19).

Another dimension of the experience is flow state, described as deep involvement in the task, intense concentration, a sense of control, fluidity of action, and altered perception of time. Flow most commonly occurs when task demands are high but remain matched to the participant's skills (21). Immersive environments may facilitate this state by reducing external distractions and providing clear goals and immediate feedback. In a cycling study, higher levels of enjoyment and flow were observed in an immersive environment than during the corresponding real-world activity (22). This does not mean, however, that every modification of exercise load will be neutral with respect to flow. Insufficient load may lead to boredom, whereas excessive resistance may disrupt movement automaticity and shift attention away from the bout toward physical sensations. Assessing flow is therefore important when designing training that combines immersion, competition, and additional resistance.

Previous studies provide evidence that additional weights or elastic bands can increase the intensity of activity in VR; however, they do not establish whether a similar effect occurs in MR during a realistic bout against an opponent controlled by artificial intelligence. In particular, there is a lack of studies comparing more than one resistance level in a mobile body-worn system while simultaneously assessing heart rate, RPE, enjoyment, and flow. Earlier experiments using elastic bands anchored externally to the body restricted freedom of movement, whereas studies involving weights used different resistance mechanisms and different applications. Moreover, MR changes the context in which the task is performed: users can see the real floor and surrounding environment, allowing them to move more naturally and maintain spatial awareness, while the digital opponent remains the central competitive stimulus.

The novelty of the present study therefore lies in the simultaneous combination of four elements: a simulated boxing bout in MR, an artificial intelligence-controlled opponent, a mobile elastic resistance system generating resistance directly on the body, and a multidimensional assessment of participant responses. The use of two resistance levels makes it possible not only to determine whether additional loading increases exercise intensity, but also to examine whether the effect is graded and whether the higher resistance level remains acceptable in terms of enjoyment and flow. Such an approach may provide practical guidance for application developers, coaches, and health-promotion specialists seeking to increase the exercise demands of immersive training without reducing its attractiveness.

The aim of this study was to assess the effects of two levels of elastic resistance applied to the upper limbs on the physiological and perceived intensity of exercise, enjoyment of the activity, and flow state in university students during a boxing bout against an artificial intelligence-controlled opponent in a mixed-reality environment. The following research questions were formulated: (1) Does the application of elastic resistance to the upper limbs increase exercise intensity assessed on the basis of the percentage of maximum heart rate (%HR_max_)? (2) Does it increase perceived exertion assessed using the Borg scale? (3) Does it affect the level of enjoyment of the activity? (4) Does it affect the level of flow state? and (5) Does the magnitude of the applied resistance differentiate the physiological and psychological responses listed above?

## 2 Materials and methods

### 2.1 Participant characteristics

The study included 36 students from the Jerzy Kukuczka Academy of Physical Education in Katowice, Poland, whose characteristics are presented in Table 1. Participants were recruited from among students of the Faculty of Physical Education. Individuals with motion sickness, hypersensitivity to flickering light stimuli, epilepsy or a history of epileptic episodes, or balance disorders were not eligible to participate. Each eligible participant had previous experience with digital entertainment in immersive virtual environments; however, none of them was familiar with the applications used in the present study. The participants also reported that their use of VR technology was occasional rather than regular. No participant reported participating in amateur or professional boxing.

**Table 1:** Characteristics of the study participants.

Variable	Overall (*n* = 36)	Women (*n* = 20)	Men (*n* = 16)
Sample size, *n* (%)	36 (100.0)	20 (55.6)	16 (44.4)
Age (years)	22.14 ± 1.48	22.25 ± 1.52	22.00 ± 1.46
Body mass (kg)	68.89 ± 13.13	62.50 ± 10.58	76.88 ± 11.74
Body height (cm)	172.28 ± 10.57	165.55 ± 6.86	180.69 ± 8.08
BMI (kg/m^2^)	23.04 ± 2.63	22.69 ± 2.63	23.48 ± 2.64
Resting heart rate (beats/min)	68.97 ± 11.64	71.95 ± 10.35	65.25 ± 12.40
Physical activity (h/week)	6.99 ± 3.41	5.73 ± 3.11	8.56 ± 3.18

The measurements were conducted at the Laboratory for Research on Health-Oriented Physical Activity at the Jerzy Kukuczka Academy of Physical Education in Katowice, in accordance with the organizational and quality procedures in force at the facility. The laboratory operates within a quality management system compliant with the PN-EN ISO 9001:2015 standard, and its current certificate is valid from December 17, 2024, to December 16, 2027. The study was conducted after obtaining approval from the Bioethics Committee of the Jerzy Kukuczka Academy of Physical Education in Katowice under Protocol No. 9/2018 of April 19, 2018, and its subsequent amendments: KB/42/2018 of July 20, 2018, and KB/27/2022 of October 25, 2022.

Before the measurements began, each participant was provided with information regarding the purpose and course of the study, the nature of the data collected, and how the data would subsequently be used. The participants were also informed that their participation was entirely voluntary and that they could withdraw at any stage without providing a reason. All individuals eligible for the study provided informed consent to participate.

The results presented in this article are part of research conducted within the grant entitled “Fighting Fire with Fire: How Active Video Games and Virtual-Reality Training Applications Can Be Used to Promote and Facilitate Health-Enhancing Physical Activity Among Adolescents and Prevent Sedentary Behaviors.” This research was funded by the Minister of Science under the “Regional Initiative of Excellence” programme, No. RID/SP/0053/2024/01, “Promoting Healthy Lifestyles and Extending Lifespan.”

### 2.2 Research environment

The simulated boxing bout was performed using the Golden Gloves VR application, run on a Meta Quest 3 headset (Meta Platforms Technologies, LLC, USA). Golden Gloves VR is a sports training application developed by Engine Room VR, designed to provide the most realistic possible representation of boxing training and competition in a digital environment. The developer presents the application as a virtual boxing platform that enables training, competition, and participation in competitive formats associated with amateur boxing. According to the available descriptions, Golden Gloves VR serves as the official esports platform of USA Boxing and the Pan American Boxing Confederation, and is intended to transfer selected elements of boxing training and sports competition to the Meta Quest headset environment.

**Figure 1:**
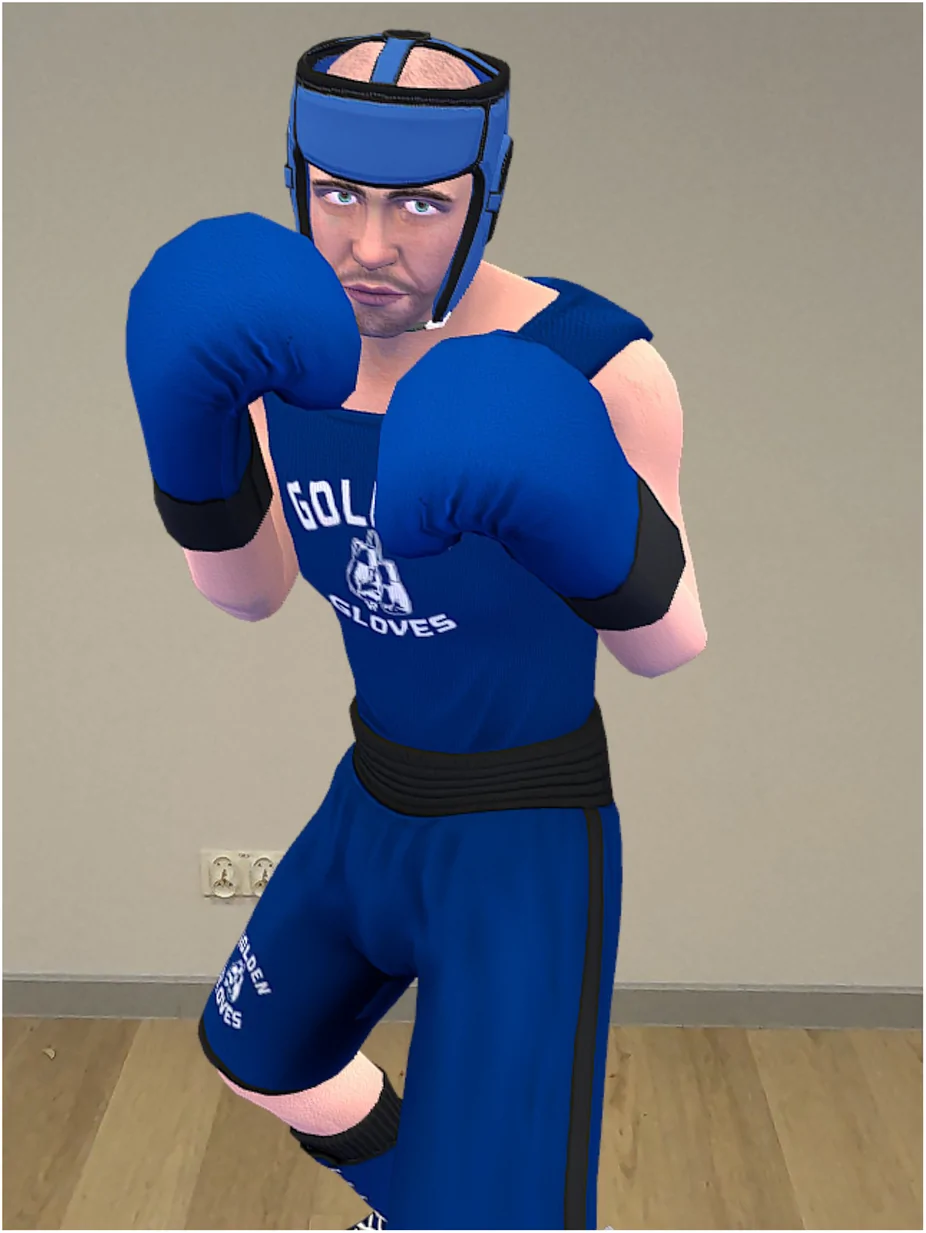
The virtual boxer from the golden gloves VR application as viewed in MR from the user's perspective.

In the present study, the application was used in mixed-reality (MR) mode rather than in fully immersive VR mode. In MR, participants viewed the actual laboratory environment through the passthrough function of the Meta Quest 3 headset, while digital elements, including the virtual opponent, were superimposed onto the view of the real-world space (Figure 1). This solution was important for participant safety because it allowed them to maintain spatial orientation within the actual room while performing dynamic boxing movements. At the same time, MR mode preserved the interactive nature of the task, as participants responded to the actions of the virtual boxer by performing movements typical of boxing with the upper limbs and trunk, including straight punches, hooks, evasive maneuvers, backward leans, movements off the line of attack, and maintaining a guard position.

The research task involved completing a boxing bout against a virtual opponent controlled by the application's algorithms. The procedure used the least demanding opponent available in the selected application mode, identified in the game as Franky. The easiest opponent was selected to standardize the exercise conditions and limit the influence of excessive task difficulty on trial performance, particularly among participants with varying levels of previous experience with boxing, active video games, or MR/VR technology. This ensured that all participants could complete the task under comparable conditions without the need to individually adjust the difficulty level of the bout. The opponent did not deliver a fixed, pre-programmed sequence of attacks. Instead, its attacks and reactions were generated dynamically in response to the participant's movements and behavior; consequently, each bout varied somewhat between participants, although all trials used the same application mode, opponent, and selected difficulty level.

The bout lasted 10 min and was performed continuously. For the purposes of the study, the duration of the trial was standardized, and the activity continued throughout the entire planned period regardless of the outcome of individual rounds or the course of the bout within the application. This meant that round outcomes, a points advantage held by either boxer, or other elements typical of the structure of a boxing bout did not interrupt the exercise exposure. The 10-minute duration was selected to provide a standardized, continuous exposure long enough for mean heart rate and the global RPE rating to reflect the cumulative exercise response, while remaining feasible in a three-condition repeated-measures protocol with 15-minute recovery intervals. The protocol was designed as sustained exergame exposure rather than as a reproduction of the formal 3-minute round structure of amateur boxing. No external cadence or prescribed punch sequence was imposed; moment-to-moment rhythm emerged from the interaction between each participant and the AI-controlled opponent. This approach ensured an identical activity duration for all participants and enabled comparisons of physiological and subjective responses across the experimental conditions analyzed.

During the trial, participants remained within the designated exercise area and performed movements determined by the course of the simulated bout. The nature of the task required repeated dynamic upper-limb movements, control of trunk positioning, responses to the opponent's attacks, and maintenance of a boxing stance. The use of Golden Gloves VR in MR mode therefore enabled the implementation of a standardized, interactive boxing exercise trial combining real-world movement within the physical space with a digitally generated opponent.

### 2.3 Research instruments and procedures

The study was conducted using a repeated-measures design. Each participant completed three 10-minute trials consisting of simulated boxing bouts performed under the following conditions: without additional resistance, with lower elastic resistance, and with higher elastic resistance. Components of the WearBands system (WearBands, USA) designed for upper-body training were used to increase the load imposed on upper-limb movements.

**Figure 2:**
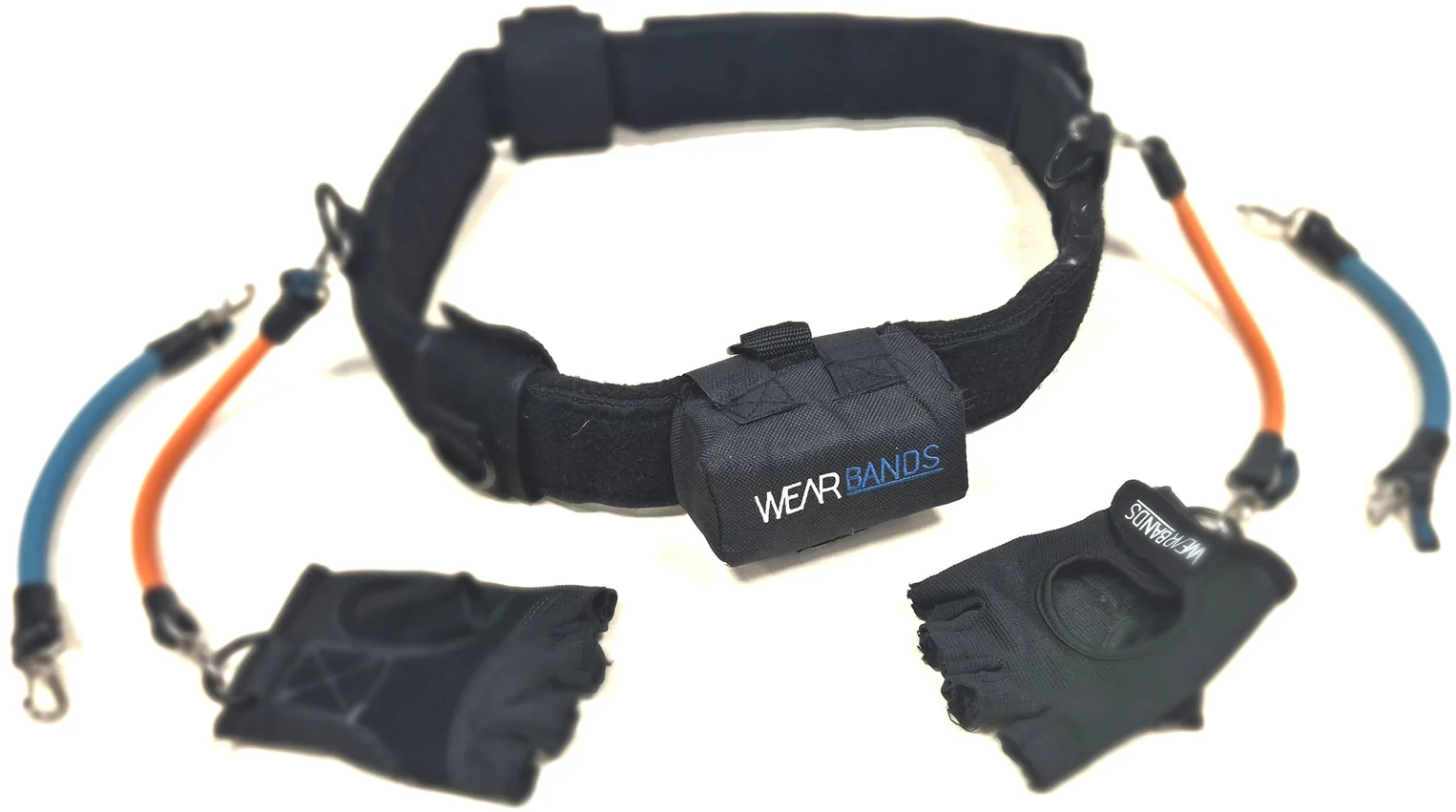
Configuration of the WearBands system used during the experimental trials.

WearBands is a body-worn elastic resistance system that enables exercise load to be increased during natural, multidirectional movements without the need to attach the elastic bands to an external anchor point. In its full configuration, the system may include a belt worn at hip level, attachment components secured to the upper limbs (gloves) and lower limbs, and elastic resistance tubes connecting the belt to these attachments. In the present study, only the hip belt, gloves, and a pair of short resistance tubes designed to load the upper limbs were used. No tubes connecting the belt to the lower limbs were applied. The components of the WearBands system used in the study are presented in Figure 2.

**Figure 3:**
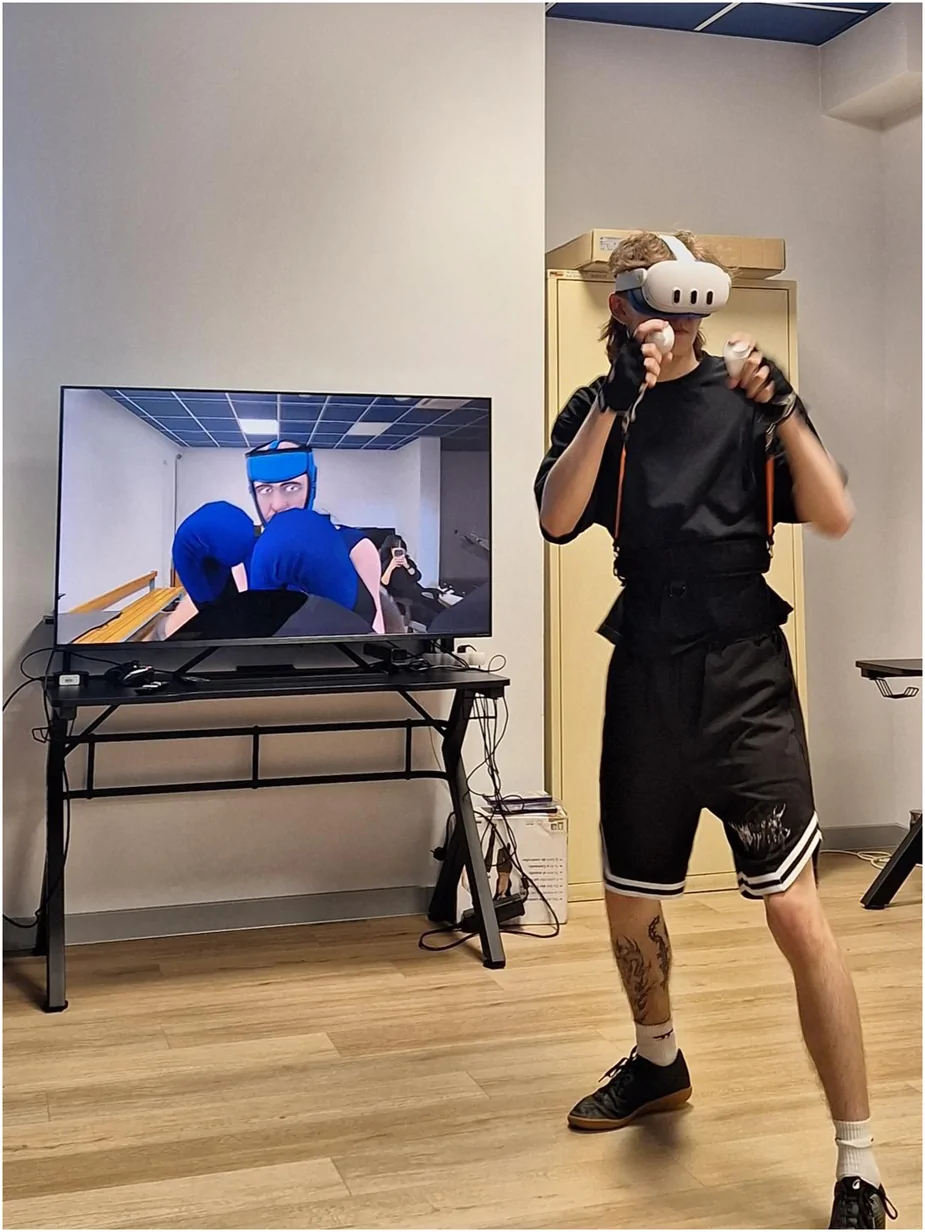
Study participant during a boxing bout against a virtual avatar using the WearBands elastic resistance system.

Each tube was attached at one end to the belt positioned at the level of the pelvis and at the other end to a glove worn on the hand. This configuration caused the tube to stretch during punch execution and when the upper limb moved away from the trunk. As the elongation of the tube increased, the resistance force acting opposite to the direction of the hand movement also increased. Thus, resistance occurred mainly during the punch execution phase, whereas during retraction of the limb the tube tension gradually decreased. The load was variable in nature and depended on the range of motion, the degree of tube elongation, and the individual punching technique. Figure 3 shows a study participant during a trial performed using the WearBands system.

Two tube variants designated by the manufacturer for upper-body training were used: orange tubes corresponding to the lower resistance level and blue tubes corresponding to the higher resistance level. The initial length of the elastic section of each tube, measured in the unstretched state, was 18 cm. The relationship between tube elongation and the generated resistance, expressed as the equivalent load in kilograms, is presented in Table 2.

**Table 2:** Resistance characteristics of the WearBands elastic tubes used during testing.

Elastic tube	25% elongation	50% elongation	75% elongation	100% elongation	125% elongation	150% elongation	175% elongation	200% elongation	225% elongation	250% elongation
Orange tube	0.77	1.47	1.78	2.26	2.52	2.8	3.15	3.36	3.64	3.96
Blue tube	1.12	1.97	2.55	3.02	3.42	3.81	4.2	4.57	4.72	5.09

Values are presented as equivalent resistance (kg).

Before the start of each trial, the symmetry of tube attachment on both sides of the body was checked to ensure that their initial length and tension were comparable for the right and left upper limbs. Participants performed the same motor tasks under all study conditions, and the only factor deliberately modified was the level of additional elastic resistance. The same tube configuration was used for all participants within each resistance condition; tube elongation was not standardized according to individual body dimensions or height and was not measured dynamically during punching.

The order of the three trials was fully counterbalanced to minimize the effects of fatigue, learning, and the order of exposure to the individual conditions. All six possible sequences were used according to the following scheme:

NR→LR→HR;NR→HR→LR;LR→NR→HR;LR→HR→NR;HR→NR→LR;HR→LR→NR,

where:

NR – no resistance;
  LR – lower resistance;
  HR – higher resistance.

After all six possible sequences had been assigned to six participants, the same allocation scheme was repeated for each subsequent group of six participants. A 15-minute rest interval was provided between trials to allow physiological parameters to return to values close to resting levels. During the rest interval, participants completed questionnaires assessing perceived exertion, enjoyment, and flow state.

Heart rate was continuously monitored during exercise using a Polar Vantage V heart rate monitor paired with a Polar H10 transmitter, which was secured to the participant's chest with an elastic strap. Individual maximum heart rate (HR_max_) was estimated using the equation 208−0.7 × age, proposed by Tanaka et al. (23). Exercise intensity was then expressed as a percentage of the estimated maximum heart rate (%HR_max_). The obtained values were interpreted in accordance with the criteria of the American College of Sports Medicine (24). Activity performed below 64% HR_max_ was classified as low intensity, activity performed at 64%–76% HR_max_ as moderate intensity, and values equal to or greater than 77% HR_max_ as vigorous intensity. With reference to the World Health Organization recommendations, activity reaching at least moderate intensity was regarded as physical activity capable of providing health benefits (25). HR and %HR_max_ were selected as practical indicators of exercise intensity in this head-mounted display-based task. Direct measurement of oxygen uptake using indirect calorimetry was not included because the required respiratory mask could interfere with comfortable headset fitting and dynamic upper-body movement and could alter the subjective experience that was also assessed. Consequently, the present measures should be interpreted as indicators of cardiovascular and perceived exercise intensity rather than direct estimates of oxygen uptake, metabolic equivalents, or energy expenditure.

Subjectively perceived exercise intensity was assessed using the 15-point Borg Rating of Perceived Exertion scale, ranging from 6 to 20 points (26, 27). Immediately after each trial, participants selected the value that best reflected their overall perception of exertion. The RPE scale was used because of its widespread application in PA research and the well-documented association between subjective ratings and physiological indicators of exercise load. Scores of 10–11 points were interpreted as corresponding to low intensity, values of 12–13 as moderate intensity, and scores ranging from 14 to 16 points as vigorous-intensity exercise (28).

Enjoyment of physical activity during the boxing trials was assessed using the short version of the Physical Activity Enjoyment Scale (PACES) (29). The questionnaire consists of eight items referring to different aspects of enjoyment and positive feelings accompanying PA. Participants responded to each statement using a 7-point Likert scale, in which 1 indicated “strongly disagree” and 7 indicated “strongly agree.” The final score was calculated as the arithmetic mean of the scores obtained for all eight items. A higher value indicated a greater level of perceived enjoyment and satisfaction with participation in a given trial.

The short version of the Flow State Scale (FSS) was used to assess flow state (30). The instrument consists of nine statements, each representing one of the core dimensions of the flow experience occurring during physical activity. Responses were provided on a 5-point Likert scale ranging from 1, “strongly disagree,” to 5, “strongly agree.” The overall score was calculated as the mean of all nine responses. A higher value indicated a stronger experience of flow while performing the task.

### 2.4 Statistical analysis

An *a priori* sample size analysis was conducted for the main within-subject effect of condition using a repeated-measures analysis of variance design with three measurements, a significance level of *α* = 0.05, and a target statistical power of 0.80. Because no directly comparable pilot data or previous studies were available to enable a reliable estimate of the expected effect size, a conventional moderate effect size was assumed (Cohen's f = 0.25). Assuming a conservative mean correlation of 0.50 among repeated measurements and a nonsphericity correction of *ε* = 1.00, the required total sample size was 28 participants. A larger number of individuals was recruited to account for the possibility of incomplete data, participant withdrawal, or the exclusion of individual measurements. The final sample of 36 participants exceeded the minimum requirement and provided adequate statistical power for the primary within-subject analysis.

Basic descriptive statistics were calculated, including means and standard deviations. The normality of the distributions was assessed using the Shapiro–Wilk test. Associations between the type of elastic resistance applied and the analyzed variables were examined using repeated-measures analysis of variance or the Friedman test, depending on whether the assumptions of parametric analysis were met. For variables analyzed using repeated-measures analysis of variance, the assumption of sphericity was assessed using Mauchly's test. If this assumption was violated, the Greenhouse–Geisser correction was planned. Effect sizes were reported as partial eta squared (η^2^p) for repeated-measures analysis of variance and Kendall's W for the Friedman test. For within-subject comparisons, the analysis of variance was supplemented with Tukey *post hoc* tests, whereas the Friedman analysis was supplemented with Durbin–Conover tests. The level of statistical significance was set at *α* = 0.05. Analyses were conducted for the entire group because sex was not a primary study factor, and dividing the sample into smaller subgroups would have reduced the statistical power of the analyses. The characteristics of women and men were presented descriptively only. Statistical analyses were performed using jamovi (version 2.2.3.0) and Statistica (version 13.3), whereas the power analysis was conducted using G*Power.

## 3 Results

### 3.1 Effects of elastic resistance on exercise intensity

The analysis showed that the use of elastic resistance applied to the upper limbs significantly affected exercise intensity during the boxing bout in MR. This applied to both mean exercise heart rate (HR) and the percentage of maximum heart rate (%HR_max_).

Mean exercise heart rate increased with the magnitude of the applied elastic resistance. The lowest value was recorded during the bout performed without resistance, in which HR was 156.61 ± 15.26 beats/min. Under the lower-resistance condition, achieved using the orange tubes, mean HR increased to 164.97 ± 13.99 beats/min, whereas under the higher-resistance condition, achieved using the blue tubes, it reached 169.75 ± 13.42 beats/min.

**Figure 4:**
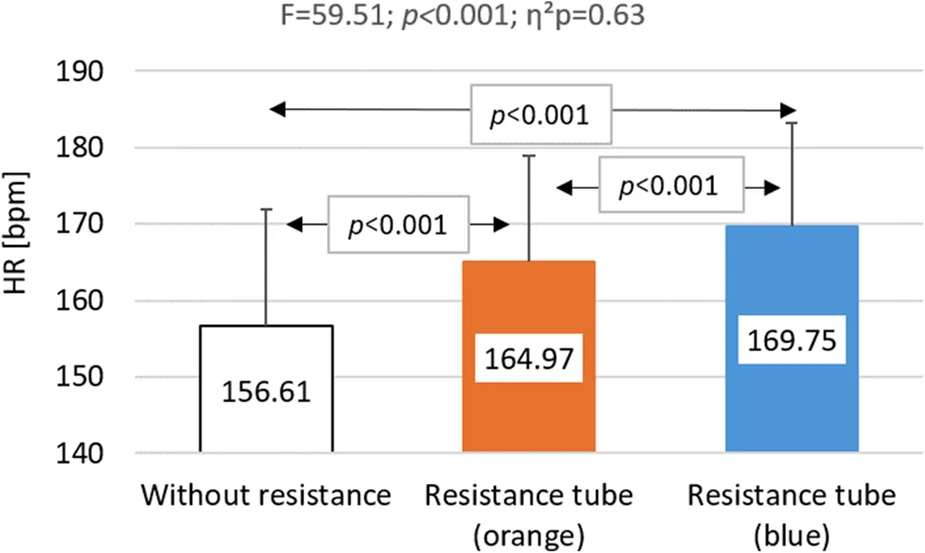
Mean exercise heart rate during the boxing bout in MR according to the applied elastic resistance. HR, heart rate; F, F statistic for repeated-measures ANOVA; η^2^p, partial eta squared; error bars, SD; horizontal arrows, *post hoc* pairwise comparisons; p, significance level.

Repeated-measures analysis of variance revealed statistically significant differences among the experimental conditions analyzed (F = 59.51; *p* < 0.001; η^2^p = 0.63). The η^2^p value, that is, partial eta squared, indicated a large effect size. *Post hoc* analysis showed significant differences between all compared conditions: between the no-resistance and lower-resistance trials, between the no-resistance and higher-resistance trials, and between the lower- and higher-resistance trials (*p* < 0.001) (Figure 4).

A similar relationship was found for %HR_max_. During the bout without resistance, the mean %HR_max_ value was 81.34 ± 7.83%. The application of lower elastic resistance increased this value to 85.68 ± 7.21%, whereas the application of higher elastic resistance increased it to 88.17 ± 6.96%.

**Figure 5:**
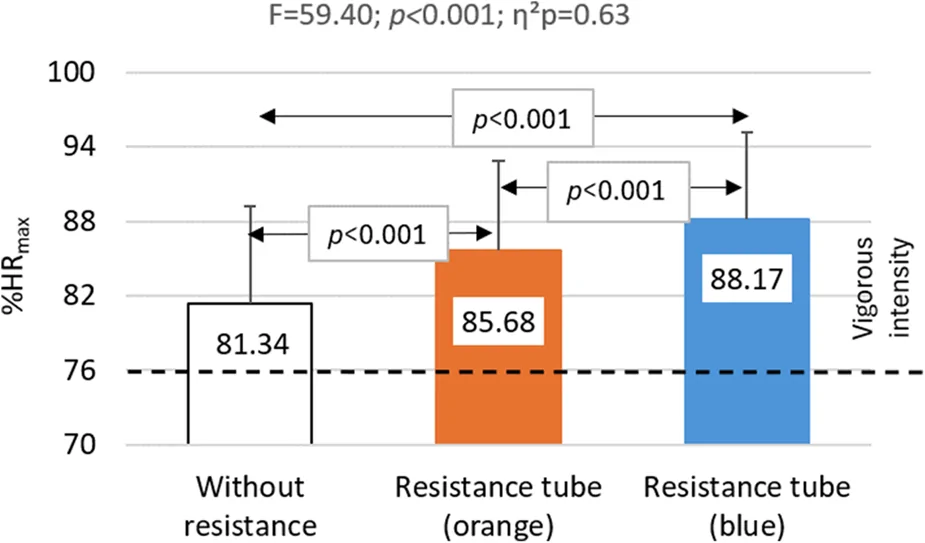
Mean percentage of maximum heart rate during the boxing bout in MR according to the applied elastic resistance. %HR_max_, percentage of maximal heart rate; F, F statistic for repeated-measures ANOVA; η^2^p, partial eta squared; dashed line, vigorous-intensity threshold; error bars, SD; horizontal arrows, *post hoc* pairwise comparisons; p, significance level.

In this case as well, repeated-measures analysis of variance revealed statistically significant differences among the study conditions (F = 59.40; *p* < 0.001; η^2^p = 0.63). *Post hoc* tests confirmed significant differences between all levels of elastic resistance (*p* < 0.001) (Figure 5).

The results indicate that the introduction of elastic resistance to the upper limbs increased the physiological intensity of the boxing bout in MR. The greater the applied resistance, the higher the heart rate indices.

### 3.2 Subjective assessment of exercise intensity

Subjective exercise intensity was assessed using the Borg 6–20 scale, in which a higher score indicates greater perceived exertion. The results showed that subjective ratings of exercise intensity also increased as the magnitude of elastic resistance applied to the upper limbs increased.

The lowest Borg scale score was recorded after the bout performed without resistance and amounted to 10.86 ± 2.32 points. Under the lower-resistance condition, the mean rating of perceived exertion increased to 13.50 ± 2.13 points, whereas under the higher-resistance condition, it reached 15.83 ± 1.84 points.

**Figure 6:**
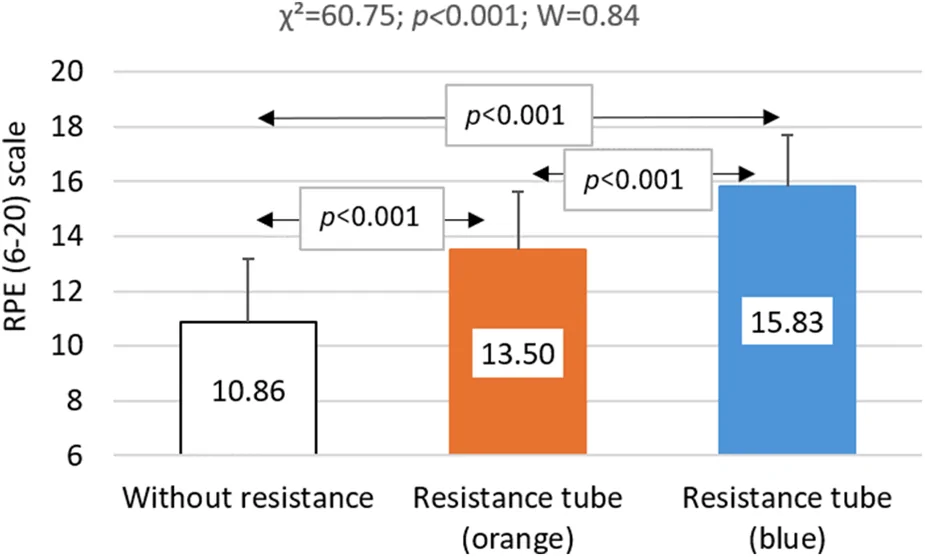
Rating of perceived exertion during the boxing bout in MR according to the applied elastic resistance. RPE, rating of perceived exertion; *χ*^,^ Friedman test statistic; W, Kendall's coefficient of concordance; error bars, SD; horizontal arrows, *post hoc* pairwise comparisons; p, significance level.

Analysis using the Friedman test revealed statistically significant differences among the analyzed conditions (*χ*^2^ = 60.75; *p* < 0.001; W = 0.84). The Kendall's W value indicated a high degree of consistency in the changes observed across participants and a strong effect of the applied resistance. *Post hoc* comparisons performed using the Durbin–Conover test showed significant differences between all trials (*p* < 0.001) (Figure 6).

This indicates that the application of elastic resistance to the upper limbs not only increased objective indicators of exercise intensity but also resulted in a marked increase in rating of perceived exertion. The highest perceived intensity was reported during the bout performed with higher elastic resistance.

### 3.3 Assessment of physical activity enjoyment

Enjoyment of the activity was assessed using the short version of the Physical Activity Enjoyment Scale (PACES). The results indicate that the application of elastic resistance to the upper limbs did not reduce enjoyment of the boxing bout in MR.

Mean enjoyment scores were very similar across all study conditions. During the bout performed without resistance, the mean score was 5.67 ± 1.08 points. Under the lower-resistance condition, the mean score was 5.72 ± 1.08 points, whereas under the higher-resistance condition, it was 5.77 ± 1.18 points.

**Figure 7:**
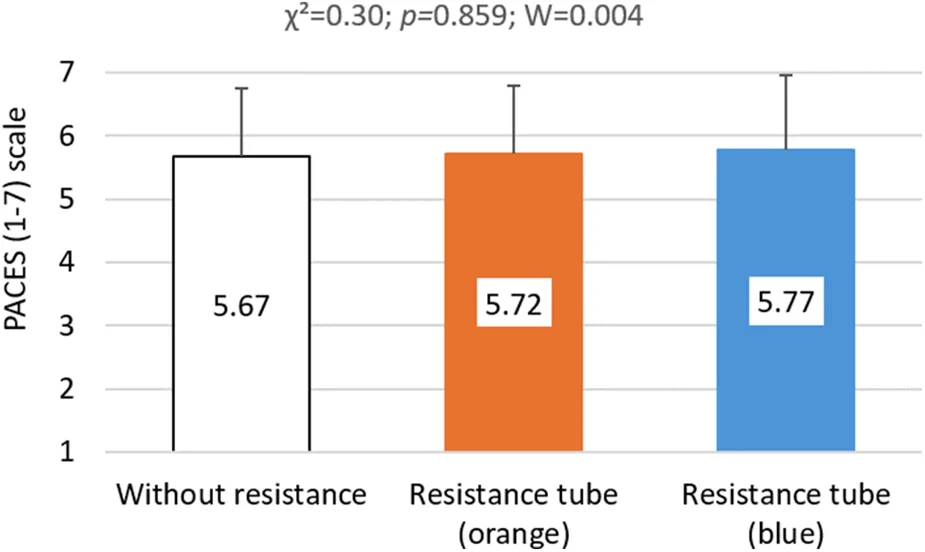
Level of enjoyment during the boxing bout in MR according to the applied elastic resistance. PACES, Physical Activity Enjoyment Scale; *χ*^2^, Friedman test statistic; W, Kendall's coefficient of concordance; error bars, SD; p, significance level.

Statistical analysis using the Friedman test revealed no significant differences among the experimental conditions (*χ*^2^ = 0.30; *p* = 0.859; W = 0.004) (Figure 7). This indicates that enjoyment remained stable regardless of whether the bout was performed without resistance, with lower elastic resistance, or with higher elastic resistance.

The results suggest that increasing exercise intensity through the application of elastic resistance to the upper limbs was not associated with a reduction in the attractiveness of the activity as perceived by the participants.

### 3.4 Assessment of flow state

Flow state was assessed using the Flow State Scale (FSS), which refers to the levels of engagement, concentration, and satisfaction accompanying the activity. The results indicate that flow levels were very similar across all analyzed conditions.

During the bout performed without resistance, the mean flow score was 4.22 ± 0.46 points. Under the lower-resistance condition, the mean score was 4.17 ± 0.49 points, whereas under the higher-resistance condition, it was 4.22 ± 0.44 points.

**Figure 8:**
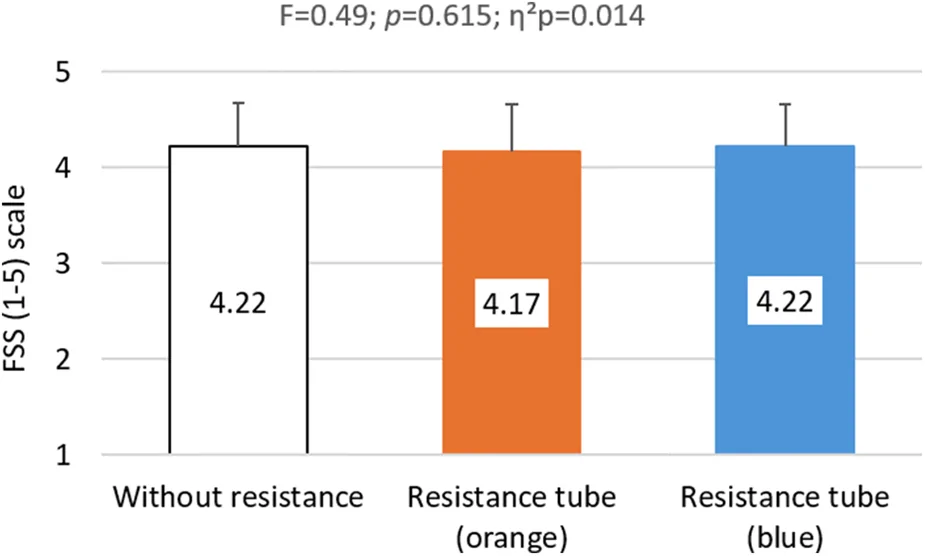
Flow state during the boxing bout in MR according to the applied elastic resistance. FSS, Flow State Scale; F, F statistic for repeated-measures ANOVA; η^2^p, partial eta squared; error bars, SD; p, significance level.

Repeated-measures analysis of variance revealed no statistically significant differences among the experimental conditions (F = 0.49; *p* = 0.615; η^2^p = 0.014) (Figure 8). The partial eta-squared value indicated a very small effect size.

The results indicate that the application of elastic resistance to the upper limbs did not significantly affect the level of flow experienced during the boxing bout in MR. Despite the higher physiological intensity and greater rating of perceived exertion, participants maintained similar levels of engagement and positive experiences associated with the activity.

## 4 Discussion

The main finding of the present study is that the use of a mobile elastic resistance system applied to the upper limbs makes it possible to progressively increase the physiological load during a simulated boxing bout in MR. Mean %HR_max_ increased from 81.34% in the no-resistance condition to 85.68% with lower resistance and 88.17% with higher resistance. A similar pattern of change was observed for mean HR and rating of perceived exertion. The large effect sizes for the heart rate indices and the high consistency of changes in RPE indicate that the observed result was not limited to a small statistical difference, but reflected a clear and consistent physiological response to increasing resistance. At the same time, enjoyment and flow state remained stable. The present data show that this acute increase did not compromise the measured short-term user experience.

Even the bout performed without additional resistance elicited vigorous-intensity exercise, while the use of elastic tubes shifted the intensity toward an even greater cardiovascular load. This finding confirms that boxing applications are among the more demanding forms of immersive active entertainment. In previous studies, the intensity of commercial VR games was strongly dependent on application mechanics. Gomez et al. (31) showed that individual active VR games may correspond to light- to moderate-intensity exercise, whereas Evans et al. (32) found considerable differences between titles, with applications involving a large proportion of squats and whole-body movements generating greater loads than games based primarily on arm movements. Sousa et al. (33), in turn, demonstrated that active games in fully immersive VR may elicit moderate- to vigorous-intensity exercise and greater engagement than sedentary experiences. Studies of FitXR boxing training have also reported significant physiological responses and more favorable psychological reactions than during comparable exercise presented on a traditional screen (8). The %HR_max_ values obtained in the present study are therefore within the upper range of those reported for active VR games.

The heart rate values obtained should also be considered in relation to the literature on the physiological demands of real-world boxing training. A systematic review with meta-analysis conducted by Finlay et al. (34) indicated that mean heart rate during simulated rounds of pad work or heavy-bag training most commonly ranges from 145 to 199 beats/min, whereas during sparring and competition it reaches approximately 180–200 beats/min (34–36). The mean heart rate recorded in the present study, ranging from 157 to 170 beats/min depending on the condition, therefore falls within the lower part of this range. This may be explained both by the selection of the least demanding artificial intelligence-controlled opponent, Franky, which was intended to standardize the trial conditions, and by the absence of real physical contact and the accompanying psychological stress characteristic of fighting a live opponent (34). It may therefore be assumed that the simulated boxing bout in MR, even without additional resistance, was comparable in intensity to pad work or heavy-bag training, which are forms of exercise commonly used in the conditioning of boxers, while avoiding the risk of injuries associated with direct physical contact.

The high intensity observed in the control condition may be attributable to the nature of the task used. Unlike applications based on striking rhythmically approaching targets, Golden Gloves requires direct responses to the behavior of an artificial intelligence-controlled opponent, including delivering punches, performing evasive movements, defending, and continuously adjusting body position. This form of bout increases the density of actions and limits passive periods during the session. Other studies have shown that the type of interface and the extent of whole-body involvement are key determinants of physiological cost. The use of an omnidirectional treadmill instead of controller-based locomotion increased exercise intensity from light to vigorous (7), while cycling games could reach moderate or vigorous intensity when the application mechanics required actual locomotor work (37, 38). These findings indicate that the health-related potential of immersive technologies should not be assessed by treating VR or MR as a uniform category, but rather in relation to the specific combination of application, control method, and additional equipment.

The observed increase in heart rate following the application of elastic resistance is consistent with previous studies on additional loading during VR activity. Polechoński et al. (16) found that wrist weights increased the intensity of Beat Saber from low to moderate without causing significant discomfort. Perrin et al. (39), in turn, showed that wrist loading during an active game increased HR, although this did not clearly translate into increased energy expenditure. In activities involving the lower limbs, additional ankle weights increased %HR_max_ during locomotion on an omnidirectional treadmill without reducing participant enjoyment (18). The common mechanism underlying these effects is an increase in muscular work demands while maintaining a similar structure of the digital task.

The most direct comparison is provided by an earlier study using the BoxVR application and elastic bands attached to the upper limbs (19). In that experiment, resistance also increased exercise intensity, but high resistance reduced enjoyment. In the present study, even the higher resistance condition did not reduce PACES scores. This difference may be attributable to the different anchoring method, resistance characteristics, type of application, and the role of resistance within the movement experience itself. Bands attached to an external anchor point may restrict changes in position, impose a fixed direction of force, and hinder the free performance of evasive movements or rotations. The WearBands system generates resistance between the hip belt and the limbs and therefore moves together with the user. This allows the mobility required during a bout to be maintained and may reduce the perception that the additional equipment interferes with control. This is a plausible explanation, although it requires direct comparison of the two attachment methods.

The gradual increase in RPE from 10.86 to 13.50 and 15.83 points was consistent with the changes in HR, indicating that participants accurately perceived the increase in exercise load. Some studies of active VR have reported discrepancies between physiological responses and subjectively perceived exertion. Stewart et al. (17) showed that, during certain games, users rated exertion as lower than suggested by their heart rate. Similarly, Glen et al. (40) found that the gaming component may improve affective responses despite a greater amount of work being performed, while Szpak et al. (41) highlighted the ability of immersive environments to distract users from some exercise-related sensations. In the present study, however, attentional distraction did not mask the substantial differences in exercise load. The higher resistance was clearly perceived, but it did not reduce enjoyment. This suggests that RPE and enjoyment are not simple opposites: users may simultaneously recognize that a task is more strenuous and still perceive it as equally enjoyable.

The stability of enjoyment deserves particular emphasis because positive affective responses are among the conditions that support the voluntary repetition of physical activity. Exergaming studies have shown that competition, feedback, and an engaging representation of movement can increase enjoyment despite greater exercise intensity. Farrow et al. (42) found that the use of VR during interval training improved performance and enjoyment, while the presence of a virtual competitor further increased exercise intensity. Monedero et al. (43) demonstrated that interactive cycling was more enjoyable and elicited greater energy expenditure than traditional cycling. Short bouts of exercise on a virtual bicycle also produced favorable psychological responses and situational motivation (38, 44). The present findings extend these observations by showing that further intensification of boxing through elastic resistance may remain psychologically neutral even when the exercise reaches a vigorous intensity.

One possible reason why enjoyment remained stable despite the higher RPE is that the additional resistance altered the physical cost of movement without changing the central game goal, feedback structure, or opponent-based interaction. In MR, passthrough allows the user to retain visual information about the room, the limbs, and the resistance system, which may support spatial control during a dynamic task (15). In addition, attention to the AI-controlled opponent and the need to react to its actions may have reduced the salience of fatigue-related sensations, as has been proposed for immersive exercise contexts (41). These mechanisms were not measured in the present study and should therefore be regarded as hypotheses rather than explanations confirmed by the data.

The absence of differences in flow state suggests that increasing the physical demands did not disturb the balance between the level of challenge and the participants’ abilities. From the perspective of flow theory, an excessive increase in difficulty could lead to a sense of overload and reduce complete absorption in the task, whereas insufficient demands could promote boredom. The high and similar FSS scores across all conditions indicate that the bouts provided a sufficiently clear goal, immediate feedback, and continuity of action, while the additional resistance primarily altered the physical cost of movement rather than the structure of the task itself. Previous studies have shown that an immersive environment may increase flow compared with comparable exercise performed in the real world (22), and that active VR may enhance the sense of challenge and engagement (33). The findings of the present experiment further suggest that flow may be relatively resistant to moderate manipulation of resistance, provided that it does not impair movement control or task effectiveness.

From an acute-exercise perspective, the WearBands system may serve as a simple tool for manipulating exercise load within a single MR session. In digital applications, the level of difficulty is usually regulated by the opponent's speed, the number of targets, the tempo of the music, or the precision required for the movement. These parameters increase perceptual-motor difficulty but do not always proportionally increase physiological load. Elastic resistance enables the physical component of the task to be dosed independently without changing the game scenario. This may be useful as research, and training approach in healthy users with different levels of physical fitness; however, its efficacy, adherence, and safety during repeated use have not been established. At the same time, the %HR_max_ values obtained in the present study indicate that higher resistance should be applied with caution. In some users, exercise intensity may approach a very high level.

Future MR exercise systems could integrate elastic resistance with AI-adaptive difficulty algorithms that use performance or wearable-sensor data to adjust task demands and external load. Such integration could enable more individualized progression than changing opponent speed or target frequency alone. However, the present study did not evaluate adaptive difficulty, real-time sensor-based coaching, or IoT implementation; these possibilities require dedicated feasibility and safety research.

The use of MR may also have facilitated the use of external equipment. Visibility of the real room, the user's own limbs, and the components of the resistance system may improve spatial control and reduce concerns about collision. Guo et al. (15) showed that passthrough in active games may improve orientation and task performance in a shared physical space. This does not mean, however, that MR automatically guarantees safety. The camera-mediated view may still involve latency or distortion, and anticipating the trajectory of a tensioned elastic tube requires caution. The novelty of the present study therefore lies not only in the application of elastic resistance during boxing, but also in combining a realistic bout against an AI-controlled opponent, a mobile resistance system, and an MR environment that preserves visual contact with the real-world surroundings.

The potential for MR exercise to be integrated into rehabilitation should be interpreted with particular caution. Although an engaging MR interface and body-worn resistance could eventually be investigated in rehabilitation programs for conditions such as Parkinson's disease or stroke, the present acute data from healthy young adults do not establish safety, feasibility, or clinical benefit in these populations. Future clinical studies should use population-specific screening, low starting loads, individualized progression, and appropriate supervision.

In summary, in a single session with young, physically active university students, applying elastic resistance to the upper limbs during an MR boxing bout increased HR, %HR_max_, and RPE without reducing measured enjoyment or flow. The mobile WearBands system may be a practical means of manipulating acute exercise load while preserving the short-term user experience. These findings do not establish long-term training effects, adherence, safety in clinical populations, or the optimal individualized resistance; these questions require longitudinal studies that include metabolic and biomechanical assessments.

### 4.1 Study limitations

The study has several limitations that should be considered when interpreting the findings. The participants were young, healthy, and physically fit university students; therefore, the results cannot be directly generalized to older individuals, less active populations, or people with health-related limitations. No participant reported participating in amateur or professional boxing, so whether the observed responses would differ in trained boxers remains unknown. The experiment involved single 10-minute sessions, which does not allow assessment of long-term training effects, adherence or the stability of enjoyment and flow state over time. Exercise intensity was determined from HR, %HR_max_, and RPE, without measurement of oxygen uptake, metabolic equivalents, or energy expenditure using indirect calorimetry. These measures were chosen as practical indicators for an HMD-based dynamic task; however, they do not directly quantify the cardiorespiratory or metabolic stimulus. The use of a respiratory mask with the headset could also have interfered with comfortable fit, dynamic movement, and the subjective experience being assessed. In addition, only one application, one type of opponent, and one resistance system were used; therefore, the results may depend on the characteristics of the solution applied. Although left–right symmetry of tube attachment was checked, tube elongation was not standardized to individual body dimensions or height and was not measured dynamically during punches. Therefore, actual resistance likely varied between participants according to body dimensions, range of motion, and movement technique. Punching biomechanics and muscle activity were not analyzed, which limits the assessment of the effects of resistance on movement technique and local loading of the upper limbs.

### 4.2 Conclusions

The use of a mobile elastic resistance system applied to the upper limbs significantly and progressively increased the intensity of a 10-minute boxing bout performed in mixed reality. As resistance increased, mean HR, %HR_max_, and RPE also increased, and the differences among all three conditions were statistically significant and characterized by large effect sizes.

The additional load did not reduce enjoyment of the activity or flow state within a single session. This indicates that the heart rate-based and perceived cost of the bout can be increased without impairing the measured short-term user experience, at least among young and physically fit university students.

Combining an MR boxing application with the WearBands system may provide an approach to acute exercise-load manipulation in MR exercise research and supervised training of healthy users. Given the high intensity achieved even without resistance and its further increase following the application of elastic tubes, resistance should be individually adjusted and introduced progressively.

Before recommendations regarding regular use can be formulated, longitudinal studies are needed to assess adherence and safety, and include measurements of oxygen uptake and energy expenditure using indirect calorimetry, analyses of punching biomechanics, and more diverse populations.

## Data Availability

The raw data supporting the conclusions of this article will be made available by the authors, without undue reservation.
